# Alopecia Areata Mimicking Frontal Fibrosing Alopecia

**DOI:** 10.7759/cureus.13361

**Published:** 2021-02-15

**Authors:** Lara Trindade de Carvalho, Nicole Izhakoff, Nekma Meah, Rodney Sinclair

**Affiliations:** 1 Dermatology, Sinclair Dermatology, Melbourne, AUS; 2 Dermatology, Herbert Wertheim College of Medicine, Florida International University, Miami, USA; 3 Dermatology, University of Melbourne, Parkville, AUS

**Keywords:** alopecia areata, frontal fibrosing alopecia

## Abstract

Alopecia areata (AA) is an autoimmune nonscarring alopecia that has variable clinical patterns, most commonly patchy disease. We report a case of AA with unusual involvement of the frontal hairline, mimicking frontal fibrosing alopecia (FFA), a form of scarring alopecia. Dermoscopic findings and response to treatment favored a diagnosis of AA. These findings highlight the major role of trichoscopy as well as the importance of including AA in the differential diagnosis for FFA.

## Introduction

Alopecia areata (AA) and frontal fibrosing alopecia (FFA) are both disorders of the hair follicle that lead to alopecia. AA is one of the most common causes of inflammation-induced hair loss, with a prevalence of 0.1% to 0.2%, affecting both children and adults as well as all colors of hair [1]. Up to one-third of patients affected are under the age of 30. This condition is associated with increased risk of autoimmune disorders (16%) such as lupus erythematosus, vitiligo and autoimmune thyroid disease [1]. AA can present in various clinical manifestations from the characteristic patches to more diffuse patterns that are harder to diagnose [2]. In contrast, FFA is a primary scarring alopecia characterized by the progressive frontotemporal recession that typically affects postmenopausal women [3]. The condition is associated with loss of follicular orifices, perifollicular erythema at the scalp margin and has an unpredictable course. Other characteristic clinical findings include total loss or general thinning of eyebrows, facial papules, glabelar red dots and depression of the frontal veins [4]. While FFA is considered irreversible, treatment of AA with corticosteroids and other therapies can lead to partial or full recovery. Therefore, it is important to distinguish between the two diagnoses.

## Case presentation

**Figure 1:**
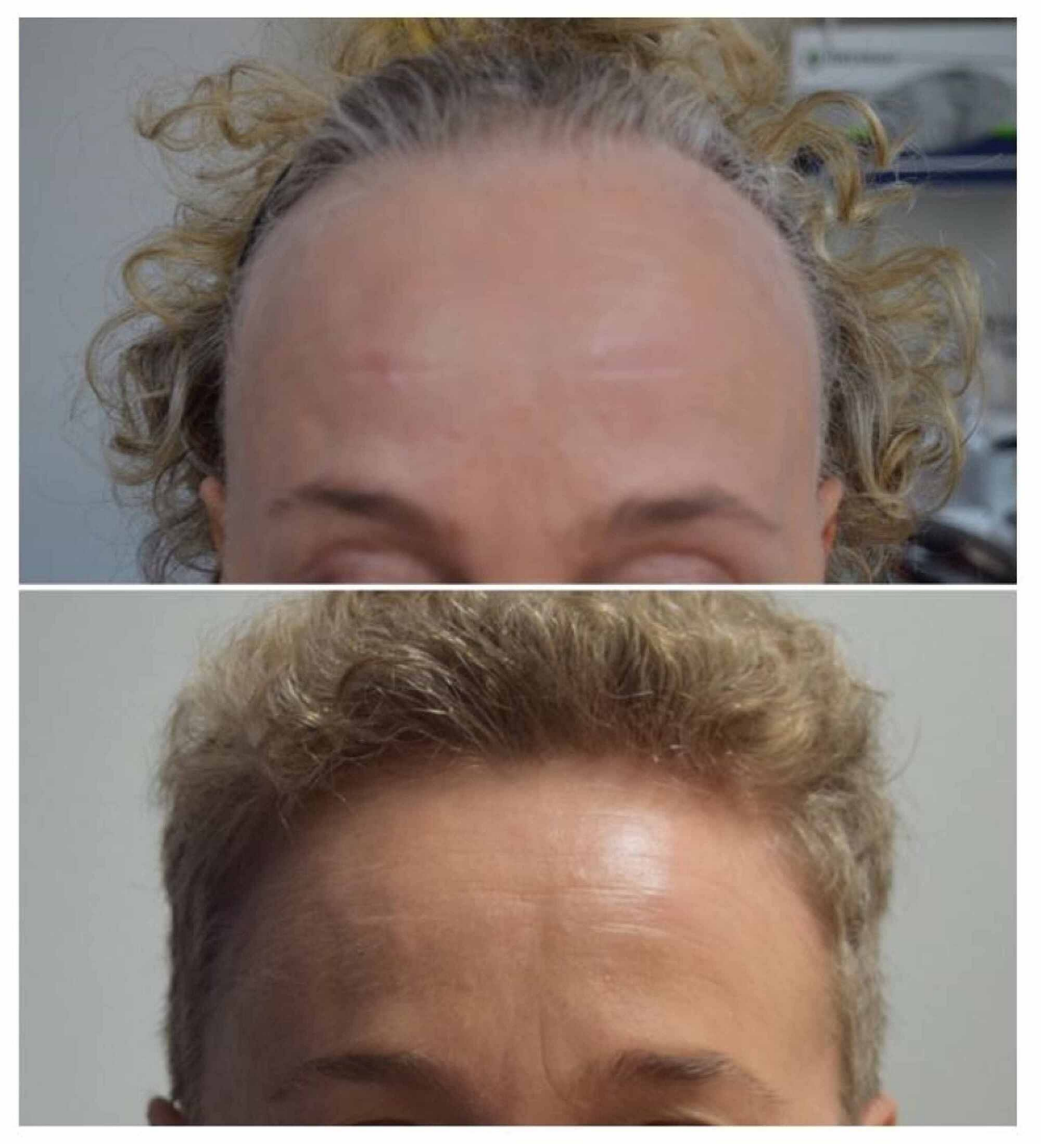
Frontal hairline before and after treatment with systemic corticosteroids.

A 59-year-old postmenopausal woman presented with a two-month history of acute shedding and associated diffuse frontotemporal hair loss (Figure 1). On clinical examination, there was no extension to the parietal‐occipital margins as seen with ophiasis AA. Hair pull test was positive along the anterior hairline. Diffuse hair thinning over the midfrontal scalp was consistent with co-existing female pattern hair loss. There was the preservation of eyebrows and eyelashes, the hair on all other body sites and her nails were normal. There was no prior history or family history of AA. Past medical history included asthma and eczema.

**Figure 2:**
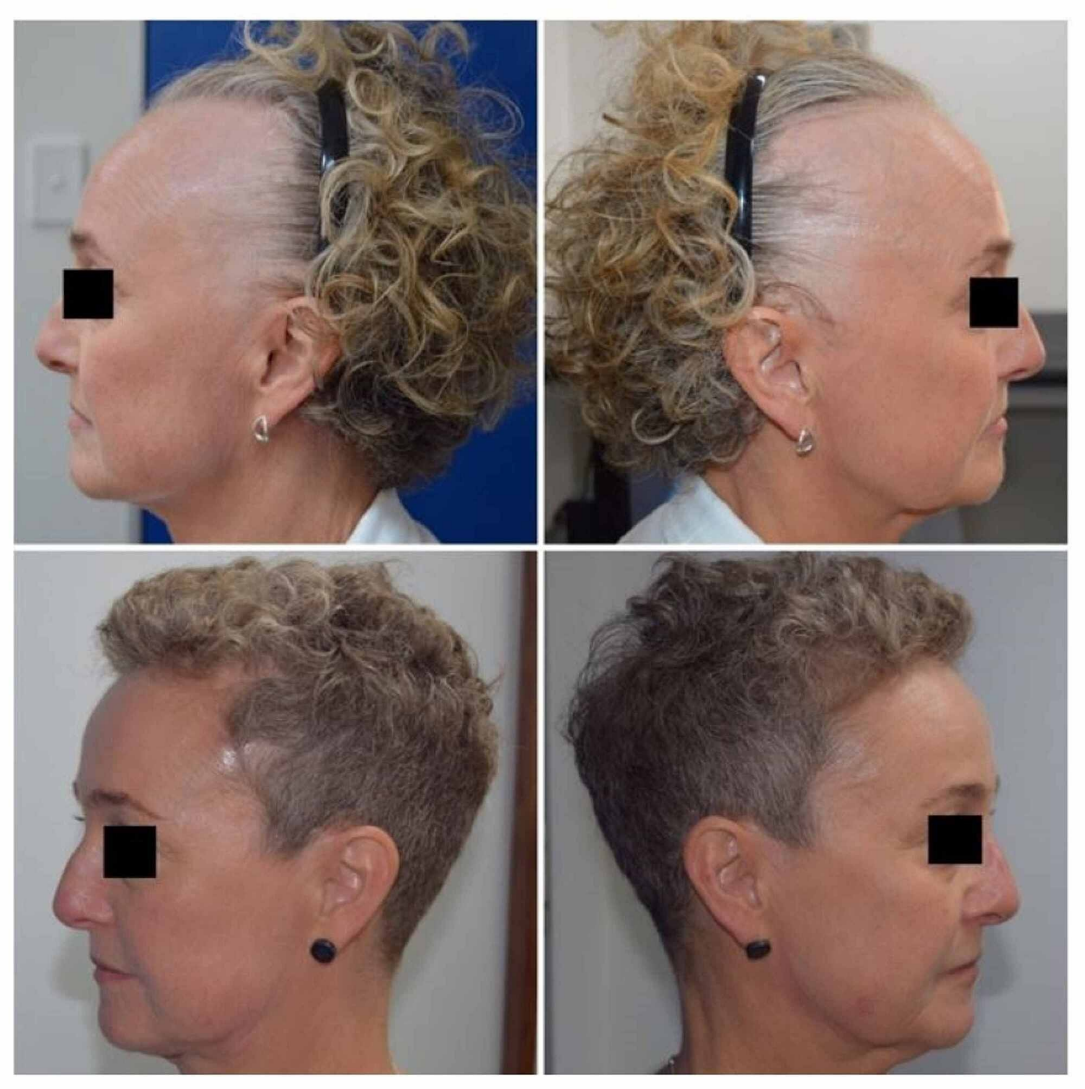
Right and left temporal areas before and after treatment with systemic corticosteroids.

Dermoscopic examination confirmed exclamation mark hairs at the edge of the alopecic area, with no evidence of perifollicular erythema and scaling. A clinical diagnosis of AA was made and a 12-week tapering course of prednisolone at 15 mg daily and minoxidil 0.25 mg daily were initiated. Six weeks later, the patient had complete terminal hair regrowth of the affected area. On her review visit eight weeks after tapering off prednisolone, she remained in remission (Figures 1, 2).

## Discussion

Clinical presentations, like our index case with an atypical distribution of AA mimicking FFA, may be challenging. Typically, cases of AA and FFA are diagnosed clinically and are easily distinguished due to their differing presentations in patient history and on physical exam. AA is more common in younger individuals and presents as smooth, round patches of normal-appearing skin [1]. FFA is more commonly seen in postmenopausal women and presents as symmetric, band-like hair loss involving the frontal hairline [3], as seen in the index patient. Whilst in AA the histological hallmark is the presence of peribulbar lymphocytic infiltrate and telogen arrest in FFA [5], the findings include fibrosing and lichenoid interface infiltrate targeting the isthmus and infundibular regions of follicles as seen in lichen planopilaris [6]. Dermoscopic findings in the index patient revealed exclamation mark hairs, a classical dermoscopic feature of AA [7]. In contrast, FFA would reveal perifollicular inflammation along the margins of the alopecic area and involvement of eyebrows, which were not present in the index case. These key findings helped us to differentiate these two conditions.

Treatment of FFA and AA both involve corticosteroids and minoxidil. However, the goal of treatment in FFA is the cessation of progressive hair loss without regrowth, whereas in AA treatment results in partial or full hair regrowth. For the index patient, timely response to systemic corticosteroids also reinforced the diagnosis of AA. Nonetheless, a scalp biopsy may be needed to establish a histopathological diagnosis in cases with ambiguous clinical presentations.

## Conclusions

Diagnosing AA can be challenging due to the various presentations. Here we presented a 59-year-old female patient with clinical features initially suggestive of FFA. Dermoscopic findings together with a timely response to systemic corticosteroids favored an atypical variant of AA. These findings emphasize the importance of including AA in the differential diagnosis for FFA. Early recognition of AA with dermoscopic evaluation is important for earlier treatment as well as patient reassurance, as this condition is treatable.
